# Amino-truncated NOV expression and its correlation with clinicopathologic features, prognosis, metastasis, and chemoresistance in bladder cancer

**DOI:** 10.1080/15384047.2024.2386753

**Published:** 2024-08-03

**Authors:** Dan Xiong, Yafei Xu, Hongbo Wang, Yunlin Ye

**Affiliations:** a State Key Laboratory of Oncology in South China, Collaborative Innovation Center for Cancer Medicine, Sun Yat-sen University Cancer Center, Guangzhou, Guangdong, China; b Medical Laboratory of the Third Affiliated Hospital of Shenzhen University, Shenzhen, Guangdong, China; c Department of Cell Biology and Genetics, Shenzhen University Health Science Center, Shenzhen, Guangdong, China

**Keywords:** Bladder cancer, nephroblastoma-overexpressed gene, amino-truncated, metastasis, chemoresistance

## Abstract

Nephroblastoma, an overexpressed gene (NOV) protein, plays an important role in proliferation, differentiation, angiogenesis, adhesion, invasion and tumorigenesis, but the function of amino-truncated NOV is different. This study is to investigate the role of amino-truncated NOV in the progression of bladder cancer. Using immunohistochemistry and Western blot analysis, we detected the amino-truncated NOV in bladder cancer, and statistical analysis was performed to estimate the association between the expression of amino-truncated NOV and the patient’s prognosis by SPSS 19.0. With transduction of amino-truncated NOV, we evaluated alteration for proliferation, migration, invasion and chemoresistance in bladder cancer cells, as well as some proteins related to Wnt/β-catenin pathway and epithelial–mesenchymal transition. The truncated variant of the NOV protein was located in a nucleus other than the cytoplasm and highly expressed in bladder cancer, which was also linked to higher pathological grade and positive lymph node metastasis as well as recurrence. The exact sequence of this truncated protein was confirmed, and it was a 26-kDa splicing. The truncated NOV protein found in bladder cancer was cut at the 187th amino acid of the full-length protein. It was also involved in bladder cancer progression and chemoresistance through a mechanism involving epithelial–mesenchymal transition (EMT) and the Wnt/β-catenin signaling pathway. Our findings provide experimental evidence that the nuclear NOV protein expression is a potential biomarker in the prognostic evaluation of bladder cancer and enhanced amino-truncated NOV expression is potentially important for bladder cancer cell invasion, metastasis and chemoresistance during progression.

## Introduction

Bladder cancer is the tenth most frequently diagnosed malignancy in the world and the thirteenth most lethal. According to the Global Cancer Incidence, Mortality, and Prevalence (GLOBOCAN) data, an additional 573,000 cases of bladder cancer were identified in 2020, which accounts for around 3% of new cancer diagnoses.^1^ At the initial diagnosis, 70% to 80% of cases are non-muscle invasive bladder cancer (NMIBC), and the remainder is muscle-invasive bladder cancer (MIBC), with 5% to 10% of patients already metastatic.^2,3^ For NMIBC, 50–70% will recur, and 10–30% will progress to MIBC, where the outcomes are not satisfactory, even though the patients have undergone radical cystectomy and chemo-radiotherapy.^4^ Neoadjuvant cisplatin-based combination chemotherapy is the standard treatment for advanced bladder cancer since the late 1980s, but it provides a median survival of only approximately 14 months for metastatic bladder cancer.^5^ Although there have been many molecular studies focused on cell proliferation, transformation, invasion, migration and chemo-resistance in bladder cancer, the mechanisms of the invasive process and the resistance to chemo-radiotherapy are still unclear.

Nephroblastoma overexpressed gene (NOV), also known as CCN3, is a member of the CCN (Cyr61, CTGF, and Nov) family. It is widely expressed in human tissues such as those of the musculoskeletal system and nervous system, as well as in blood vessels. Recent studies have shown that the secreted NOV protein plays an important role in proliferation, differentiation, angiogenesis, adhesion, invasion and tumorigenesis.^6,7^ NOV acts as an oncogene in cervical cancer, melanoma, bladder cancer and renal cell carcinomas, while in breast cancer, chronic myelogenous leukemia (CML) and colorectal cancer, it plays an anti-oncogenic role.^8–14^ Interestingly, the role of NOV in prostate cancer has been controversial.^15–17^ A growing body of evidence supports the presence of an amino-truncated variant of NOV in different cells. An amino-truncated nuclear NOV protein containing the carboxy-terminal (CT) module was revealed by western blot analysis in HeLa and 143 osteosarcoma cancer cells.^18,19^ Nuclear NOV proteins were detected by immunofluorescence in several human cancer cell lines, including HeLa, osteosarcoma 143, choriocarcinoma Jeg3, hepatocellular carcinoma cells, melanoma, and rat C6 glioma cells, as well as in some cases of osteosarcomas and Ewing’s sarcomas.^19–26^ NOV proteins deprived of a signal peptide are directed to the nucleus, and the C terminal module of NOV is responsible for the nuclear localization of NOV that is lacking signal peptide.^27^ A 32-kDa amino-truncated form is also found both in a secreted form and in the nucleus of different cell types.^19^ This short NOV isoform has been described as lacking the N-terminal domain, as determined by reactivity with the K19M antibody recognizing the C terminal domain. Amino-truncation of NOV has been associated with oncogenic transformation and cancer development.^20,22^

Here, we provide the first evidence that a 26-kDa truncated variant lacking the N-terminal domain exists in bladder cancer. Meanwhile, we show that the amino-truncated NOV has a high expression level in bladder cancer, which is also linked to a higher pathological grade and positive lymph node metastasis, as well as to recurrence. Moreover, it is overexpressed in muscle-invasive and migratory bladder cancer cells. We have assessed the functional role of the amino-truncated NOV protein in bladder cancer cells. Our analysis of NOV protein expression points to its functional role in bladder cancer progression and chemo-resistance through a mechanism involving epithelial–mesenchymal transition (EMT) and the Wnt/β-catenin signaling pathway.

## Materials and methods

### Patients and tissue specimens

For real-time PCR and western blot analysis, 16 paired bladder cancer and adjacent normal bladder tissues were collected from patients who underwent treatment from January 2011 to May 2012. Besides, 204 paraffin-embedded specimens of bladder cancer were collected between January 2000 and December 2010 for immunohistochemical assay. There were 20 female patients and 184 male patients, with ages ranging from 15 to 87 y (median age 60.9 y). For these patients, trans-urethral resection of bladder tumor (TURBT) and intravesical chemotherapy were performed for non-muscle invasive bladder cancers and radical cystectomy was performed for muscle-invasive ones. Histologic grade was determined according to the WHO 1973 guidelines, and clinical staging was performed according to the American Joint Committee on Cancer (AJCC) TNM Staging System (7th edition, 2010). The clinicopathological characteristics of bladder cancer are summarized in Table 1. All the specimens had been clinically and histologically diagnosed by the Sun Yat-sen University Cancer Center (Guangzhou, China). Prior informed consent from the patients and approval from the Sun Yat-sen University Cancer Center Institute Research Ethics Committee (approval number: YP2008063) were obtained before using these clinical materials.

**Table 1.** Correlation between the cytoplasm expression of NOV protein and clinical pathologic factors in bladder cancer.

Characteristics	N	NOV cytoplasm expression		*P* value
		Low	High	
**Gender**				0.197
Male	184	56(30.34%)	128(69.56%)	
Female	20	3(15%)	17(85%)	
**Age**				0.355
<65	110	35(31.82%)	75(68.18%)	
>65	94	24(25.53%)	70(74.47%)	
**Tumor number**				0.632
≤3	128	39(30.47%)	89(69.53%)	
>3	76	20(26.32%)	56(73.68%)	
**Tumor size**				0.019
≤3	85	17(20%)	68(80%)	
>3	119	42(35.29%)	77(64.71%)	
**Histological grade**				0.02
G1+G2	96	20(20.83%)	76(79.17%)	
G3	108	39(36.11%)	69(63.89%)	
**Clinical stage**				0.001
Ta, T1	106	16(15.09%)	90(84.91%)	
T2、T3、T4	98	43(43.88%)	55(56.12%)	
**Lymph node**				0.001
N0	178	44(24.72%)	134(75.28%)	
N1	26	15(57.69%)	11(42.31%)	
**Status**				0.642
Recurrence-free	93	25(26.88%)	68(73.12%)	
Recurrence	111	34(30.63%)	77(69.37%)	

### Immunohistochemistry analysis, real-time PCR, Western-blot, immunofluorescence analysis, N-Terminal microsequencing and casein zymography

These analyses were performed as shown in supplementary data.

### Follow-up and clinical outcomes assessment

The overall survival time (OS) was defined as the time between surgery and the last follow-up time or date of death, and disease-free survival (DFS) was defined as the time between surgery and next tumor recurrence/progression. In 204 patients, 113 of them underwent bladder-sparing operation. The cystoscopy was performed every 3 months for the first 2 y, every 6 months for 2–4 y, and then annually in the 72 patients after the treatment. The other 91 patients, who underwent radical cystectomy, did the abdominal and pelvic CT every 3 months for the first 2 y, every 6 months for 2–4 y, and then annually. The recurrence was defined as histopathologic findings with abnormal bladder mucosa or tissue of bladder cancer postoperatively. For 106 non-muscle invasive bladder cancer (NMIBC) patients who were treated with intravesical chemotherapy, the chemotherapy failure was defined as bladder recurrence or progression.

### Cell lines, plasmids and stable transduction

Human bladder epithelial cell line BLN1 was grown in keratinocyte serum-free medium (KSF) (Invitrogen Corp, Carlsbad, CA) supplemented with epidermal growth factor. The human bladder cancer cells T24, Biu87, EJ and 5637 were obtained from the American Type Culture Collection (ATCC, Rockville, MD) and cultured in RPMI 1640 Medium (GibcoTM, Fisher Scientific Corp, Leicestershire, UK) containing 5% fetal bovine serum (FBS) (Invitrogen Corp, Carlsbad, CA). The Sf9 and Hi-5 cell lines were adherent cultured at a temperature of 26°C–28°C, and CO_2_ exchange was not required. The pH value of the medium was 6.1–6.4, the osmotic pressure was 345–380mOsm/kg, and the dissolved oxygen saturation was 10–50%.

The pMSCV-NOV^188-356aa^ construct was generated and transfected into Biu87, EJ and 5637 cell lines according to the instructions. pMSCV plasmid was used as the control. Retroviruses were produced by transient transfection according to the manipulate method (Invitrogen Corp, Carlsbad, CA).^28^ All retrovirally infected cells were maintained under 1.0 µg/ml Puromycin selection and used as stable cells.

### Cell proliferation, wound-healing and migration assays

The viability of cells was evaluated by Cell Counting Kit-8 (CCK8, Dojindo Molecular Technologies, Japan) according to instructions. Tumor cells were seeded onto 96-well plates at a density of 4 × 10^3^ cells per well. CCK8 solution was added to each well at the time points of 24 h, 48 h, 72 h, and 96 h. The plates were examined at 450 nm absorption with a microplate reader after incubating for 4 h at 37°C.

For wound-healing analysis, cells were trypsinized and seeded equally into 6-well plates to grow to almost full confluence in 24 h, followed by non-serum starvation for another 24 h. The cell monolayer was subsequently scratched with a sterile 100 μL pipette tip. After scratching, the cells were washed with PBS and then cultured in a serum-free medium. Cell migration images were captured at time points of 0 h and 48 h by an inverted microscope (100X).

For the transwell migration assay, 1.5 × 10^5^ cells in 200 μL of serum-free 1640 were added to the cell culture inserts with an 8-μm microporous filter without extracellular matrix coating (Becton Dickinson Labware, Bedford, MA). The 1640 medium containing 10% FBS was added to the bottom chamber. After 36 h of incubation, the cells on the lower surface of the filter were fixed and stained prior to microscopic examination. The number of migrated cells in three random optical fields (100 X) for each filter from triplicate filters was averaged.

### Chemoresistance analysis

Bladder cancer cell lines 5637 and T24 were seeded into 96-well plates (Becton Dickinson, Franklin Lakes, NJ) overnight. The cisplatin stock solution (10 mM) was prepared in PBS. DDP was diluted in culture media (0, 1, 2, 4, 6, 8, 12 μmol/L) and added to the plates, then incubated for an additional 48 h. The control group contained 0.2% PBS. Cell viability assays were performed according to the manufacturer’s standard protocol. The absolute 50% inhibitory concentrations (IC50) were calculated according to Seabaugh, and dose–response curves were produced with GraphPad Prism 5 software (GraphPad Software Inc., La Jolla, CA).

### Statistical analysis

All the statistical analyses were performed using the SPSS 19.0 software (SPSS Inc, Chicago, IL, USA) and GraphPad Prism 5 (GraphPad Software Inc., La Jolla, CA) software. The paired T-test was used to analyze the significance of NOV mRNA levels in the paired samples. The chi-square test was utilized to analyze the relationship between NOV expression and clinic-pathological factors. Kaplan – Meier and log-rank tests were calculated for survival analysis. Multivariate Cox regression analysis was performed for all variables which were found to be significant by univariate analysis. All *p* values were calculated using two-sided tests, and *p* values less than 0.05 were considered to be statistically significant.

## Results

### Detection of NOV mRNA and protein expression in bladder tissues

**Figure 1.**
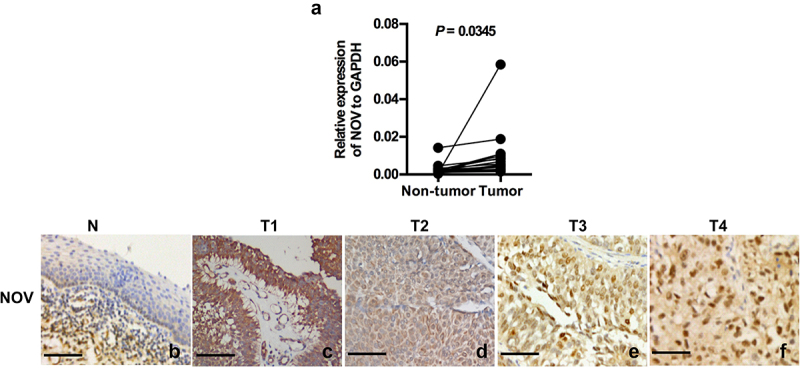
The mRNA and protein of NOV expression in bladder cancer and normal tissue. (a). Upregulated mRNA level of NOV in 16 paired bladder cancer and adjacent normal urothelium b-f. The protein expression of NOV was higher in bladder cancer tissues (c–f) than in normal bladder urothelium (b), and NOV was expressed mostly in the cytoplasm for T1-2 cancer tissues (c–d) and nuclear for T3-4 cancer tissues (e–f) using immunohistochemical method. Scale bars, 200 µm. (b): normal tissue; (c): T1 bladder cancer tissue; (d): T2 bladder cancer tissue; (e): T3 bladder cancer tissue; (f): T4 bladder cancer tissue.

Real-time PCR was performed to investigate the levels of NOV mRNA in bladder cancer and adjacent normal tissues. In 16 paired samples extracted from cystectomy specimens, the expression of NOV was up-regulated in bladder cancer tissue compared to expression levels in the adjacent normal urothelium (*p* = .0345) (Figure 1(a)).

The expression and subcellular location of the NOV protein were investigated by immunohistochemistry in 204 paraffin-embedded, archival bladder cancer tissues and 10 specimens of adjacent normal bladder tissues. The normal bladder tissues did not express the NOV protein (Figure 1(b)). The NOV protein staining was localized mainly in the cytoplasm of all tumor tissues (Figure 1(c)), while a total of 175 of 204 (85.7%) bladder cancer samples showed the NOV protein in the nucleus. Moreover, the nuclear accumulation of NOV protein staining was distinct in muscle-invasive bladder cancer (Figure 1(d–f)). Based on the staining intensity, the positive expression of the NOV protein in the nucleus was subdivided into none (Figure 1(b–c)), weak (Figure 1(d)), median (Figure 1(e)) and strong (Figure 1(f)). The negative staining is shown in Figure 1(b). The low nuclear expression of NOV included both weakly positive and negative staining. The median positive and strongly positive staining was considered to represent high nuclear expression levels of NOV. Based on the same rules, the cytoplasmic expression of NOV was also assessed both in the bladder cancer tissue and adjacent normal bladder tissues.

### Relationship between the cytoplasmic and nuclear expression levels of NOV and clinicopathological features

The relationship of clinical characteristics with the cytoplasmic and nuclear expression of NOV was evaluated. Intense expression of cytoplasmic NOV in bladder cancer was not significantly correlated with gender, age, tumor number or recurrence (*p* > .05) but was significantly correlated with tumor size, clinical stage, histopathological grade and lymph node metastasis (*p* < .05) (Table 1). The relationship between the nuclear expression of the NOV protein and clinical characteristics is shown in Table 2. Strong expression of nuclear NOV was significantly correlated with the histopathological classification of patients, T stage, lymph node metastasis and recurrence (*p* < .05), while it was not correlated with age, gender or tumor size (*p* > .05). The higher the pathological T stage and the histopathological classification were, the stronger the nuclear NOV expression (*p* = .001). In patients with higher histopathological classifications (G3) and muscle-invasive bladder cancer (T2-T4), the nuclear expression level of NOV was higher. We noted that in muscle-invasive bladder cancer the intensity of expression of nuclear NOV was higher than the intensity of expression in non-muscle-invasive bladder cancer (80.61% vs. 40.57%, *p* = .001). In patients with low histopathological classifications (G1+G2), the intensity of the nuclear NOV expression was lower than the intensity in patients with high histopathological classification (G3) (44.79% vs 73.15%, *p* = .001). There was a significantly higher recurrence in the group with high expression levels of nuclear NOV protein (68.47%) than in the group with low expression levels of nuclear NOV protein (31.53%) (*p* = .007).

**Table 2.** Correlation between the nuclear expression of NOV protein and clinical pathologic factors in bladder cancer.

Characteristics	N	NOV nuclear expression		*P* value
		Low	High	
**Gender**				0.64
Male	184	73(39.67%)	111(60.33%)	
Female	20	9(45%)	11(55%)	
**Age**				0.116
<65	110	50(45.45%)	60(54.55%)	
>65	94	32(34.04%)	62(65.96%)	
**Tumor number**				0.661
≤3	128	53(41.40%)	75(58.59%)	
>3	76	29(38.16%)	47(61.84%)	
**Tumor size**				0.06
≤3	85	41(48.24%)	44(51.76%)	
>3	119	41(34.45%)	78(65.55%)	
**Histological grade**				0.001
G1+G2	96	53(55.21%)	43(44.79%)	
G3	108	29(26.85%)	79(73.15%)	
**Clinical stage**				0.001
Ta, T1	106	63(59.43%)	43(40.57%)	
T2、T3、T4	98	19(19.39%)	79(80.61%)	
**Lymph node**				0.001
N0	178	79(44.38%)	99(55.62%)	
N1	26	3(11.54%)	23(88.46%)	
**Status**				0.007
Recurrence-free	93	47(50.54%)	46(49.46%)	
Recurrence	111	35(31.53%)	76(68.47%)	

### Nuclear expression of NOV predicts poor prognosis of bladder cancer

**Figure 2.**
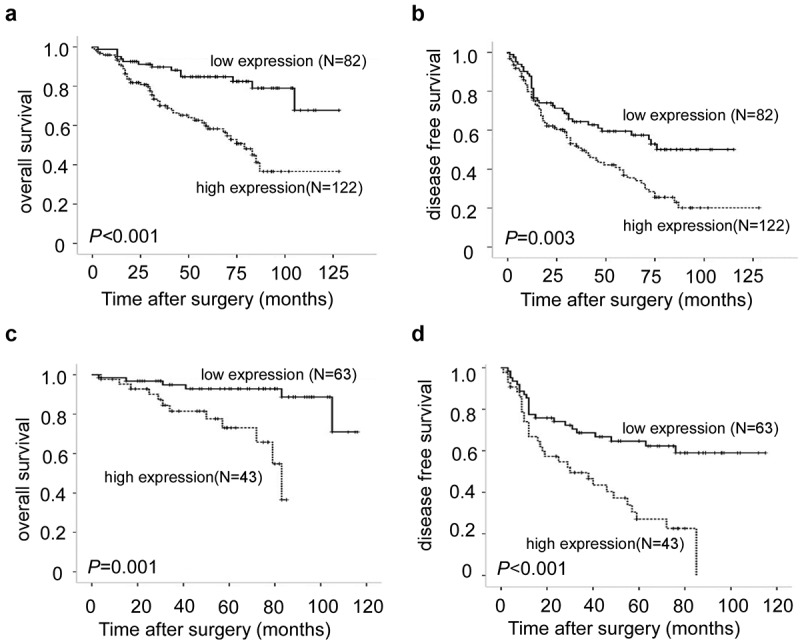
Kaplan-Meier analysis of overall survival and disease-free survival in bladder cancer patients. (a): overall survival analysis of 204 bladder cancer patients with different nuclear NOV protein expression. (Log-rank test: *p* < .001). (b): disease-free survival analysis of 204 bladder cancer patients with different nuclear NOV protein expression. (Log-rank test: *p* = .003). (c): overall survival analysis of 106 non-muscle invasive bladder cancer patients with different nuclear NOV protein expression. (Log-rank test: *p* = .001). (d): disease-free survival analysis of 106 non-muscle invasive bladder cancer patients with different nuclear NOV protein expression. (Log-rank test: *p* < .001).

Of the total 204 patients, 63 (30.9%) patients died after the treatment of bladder cancer. The patients’ overall survival time was significantly correlated with the expression level of the nuclear NOV in bladder cancer tissue (*p* = .001, Figure 2(a)). There was a significantly lower 5-y overall survival rate in the group with high expression levels of nuclear NOV protein (59.8%) than in the group with low expression levels of nuclear NOV protein (82.9%). Meanwhile, the cumulative 5-y disease-free survival rate (DFS) rate was 37.7% in the high nuclear NOV expression group, whereas it was 57.3% in the low nuclear NOV expression group (*p* = .003, Figure 2(b)). In the univariate and multivariate Cox regression analyses, the high nuclear expression of NOV was an independent prognostic factor of overall survival (95% confidence interval: 0.244–0.946; *p* = .034), and clinical T stage and N stage were also two independent and significant prognostic factors for overall survival *(p* = .029 and 0.023, respectively) (Table 3). However, the low cytoplasmic NOV expression was not an independent prognostic factor for overall survival (*p* = .244). Thus, our data indicated that the nuclear expression of NOV is a potential marker for the prognosis of bladder cancer.

**Table 3.** Univariate and multivariate analysis of potential factors for overall survival in bladder cancer (*n* = 204).

Factors	Category	Univariate	Multivariate		
		*P* value	Wald value	Hazard ratios (95% CI)	*P* value
Age	≥65 vs < 65	0.343			
Sex	Male vs Female	0.303			
Size	≥3 vs < 3	0.079	0.041	0.944 (0.539–1.654)	0.840
Number	≥3 vs < 3	0.106			
T stage	TaT1 vs T2-4	0.001	4.767	0.465 (0.234–0.925)	0.029
Lymph node	N0 vs N1	0.001	5.162	0.439 (0.216–0.893)	0.023
Grade	G1-G2 vs G3	0.020	0.150	1.123 (0.624–2.020)	0.699
Cytoplasm NOV	Low vs High	0.004	1.357	0.721 (0.416–1.250)	0.244
Nuclear NOV	Low vs High	0.001	4.491	0.480 (0.244–0.946)	0.034

CI: confidence interval.

It is interesting to note that of the 204 cases included, 106 patients had non-muscle-invasive bladder cancer who were primarily treated with TURBT and intravesical chemotherapy, and 53 recurrent diseases were observed. The nuclear NOV value in the subgroup with NMIBC was analyzed separately. In the survival analysis, we found that the higher the level of nuclear NOV expressed, the worse the overall survival (OS) rate and DFS rate of patients (*p* = .01 and 0.001, Figure 2(c–d)). Furthermore, in the univariate and multivariate analyses of disease-free survival, the nuclear NOV protein level was also an independent risk factor for subsequent recurrence (95% confidence interval: 0.244–0.770, *p* = .004), while low cytoplasmic NOV expression was not a prognostic factor (*p* = .786, Table 4). Therefore, the high nuclear NOV expression may be a predictive marker for the risk of recurrence in NMIBC patients.

**Table 4.** Univariate and multivariate analysis of potential factors for disease-free survival in non-muscle-invasive bladder cancer (*n* = 106).

Factors	Category	Univariate	Multivariate		
		*P* value	Wald value	Hazard ratios (95% CI)	*P* value
Age	≥65 VS < 65	0.430			
Sex	male vs female	0.517			
Size	≥3vs < 3	0.430			
Number	≥3vs < 3	0.066	1.831	0.686 (0.397–1.184)	0.176
Grade	G1-G2 vs G3	0.134	0.421	0.829 (0.470–1.462)	0.517
Cytoplasm NOV	Low vs High	0.786			
Nuclear NOV	Low vs High	0.001	8.121	0.433 (0.244–0.770)	0.004

CI: confidence interval.

### The identification and verification of the amino-truncated form of NOV in bladder cancer

**Figure 3.**
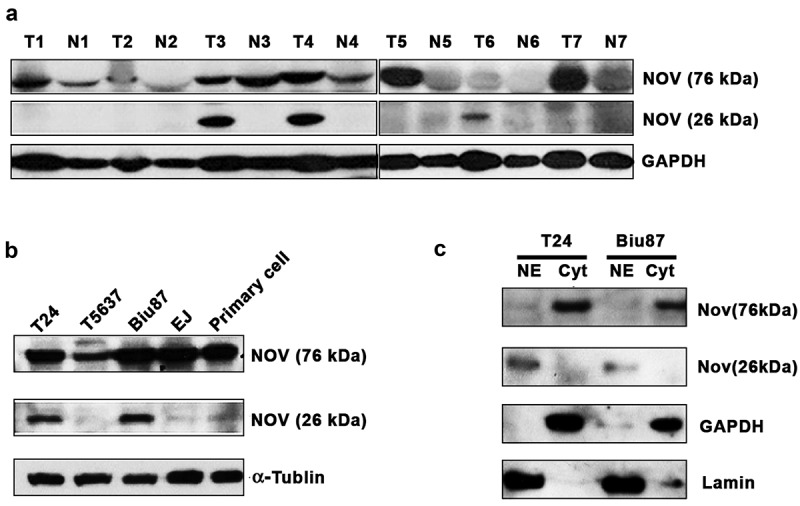
The expression of amino-truncated NOV in bladder cancer. (a): the expression of amino-truncated NOV (NOV^188–356^) in 7 paired bladder cancer and adjacent normal urothelium (b) the expression of amino-truncated NOV (NOV^188–356^) in bladder cancer cell lines (c): the location of amino-truncated NOV (NOV^188–356^) expressed in bladder cancer cells.

Amino-truncated NOV proteins were previously detected in MAV-induced nephroblastoma cells, in the nuclei of HeLa and 143 osteosarcoma cells, in normal brain tissues, and other tissues. Our clinical data indicated that NOV might exist in nuclear and cytoplasmic forms in bladder cancer. The protein expression of NOV in seven paired bladder tissues was detected using western blot analysis. We found that the spliced variant of NOV existed in primary bladder cancer tissues (Figure 3(a)). Moreover, the expression of the spliced variant only existed in advanced bladder cancer tissue. We also found that the expression level of the spliced NOV variant was higher in cell lines T24 and Biu87 from the malignant stage of bladder cancer than its level in the bladder cancer cell lines 5637 and EJ from NMIBC (Figure 3(b)). Following verification, a nuclear and cytoplasmic extraction kit was used to separate the nuclear and cytoplasmic components of the bladder cancer cell lines T24 and Biu87. We found that the spliced variant was expressed in the nuclei of T24 and Biu87, while the full-length form of NOV was expressed in the cytoplasm of bladder cancer cells (Figure 3(c)).

**Figure 4.**
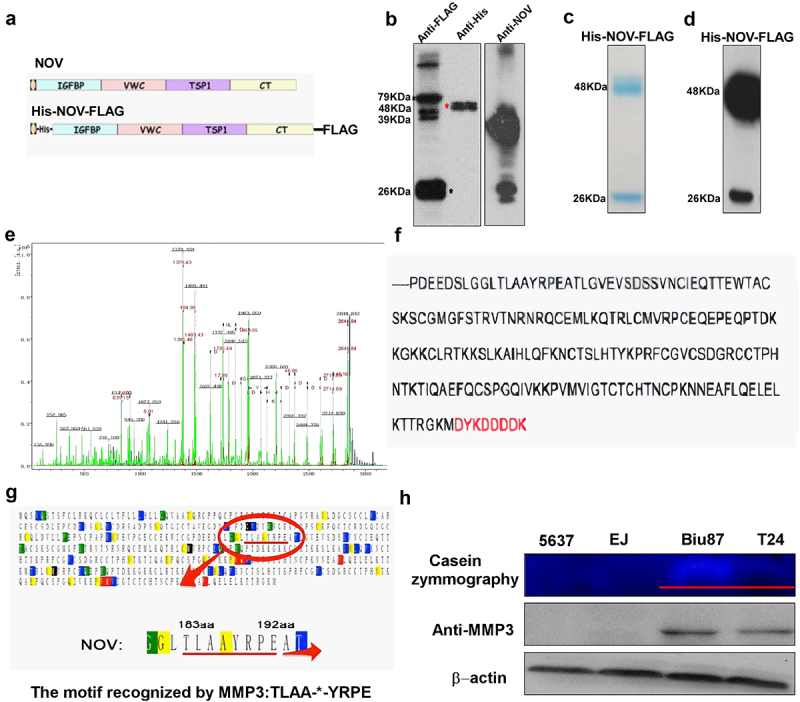
The verification of the amino-truncated NOV in bladder cancer. (a–b): bac-to-bac baculovirus expression system of NOV protein (c–d): purification of NOV protein (e–f): results of matrix-assisted laser desorption ionization-time of flight mass spectrometry (g)–(h): the correlation between MMP3 and amino-truncated NOV (NOV^188–356^).

Then, we expressed and purified the NOV protein in large quantities through the Bac-to-Bac baculovirus expression system (Figure 4a–d). By applying matrix-assisted laser desorption ionization-time of flight MS to the purified NOV variant, the accurate molecular weight was 21,473 Da, and this result implied that the N-terminal was at 187–191 AA. Combined with information from reported studies, and N-terminal microsampling technology confirmed the 187AA.

We determined the exact sequence of this truncated protein and found that the truncated NOV protein is cut at the 187th amino acid of the full-length protein found in bladder cancer (Figure 4(e)). Accordingly, we analyzed and predicted the enzyme involved in the truncation of the protein via Prosper, the enzyme database. The prediction by Prosper showed that MMP3 could recognize the motif -TLAA-*-YRPE- around the truncated point (between the 185th and 192nd amino acids), which suggested the involvement of this enzyme in forming the truncated NOV (Figure 4(f,g)). We examined the enzymatic activity and the expression of MMP3 using casein-zymography and western blot analysis, respectively. The enzymatic activity and the expression of MMP3 were higher in cell lines T24 and Biu87 from the malignant stage of bladder cancer than they were in bladder cancer cell lines 5637 and EJ from NMIBC (Figure 4(h)). These results indicated that the enzymatic activity and the expression of MMP3 were very important for the formation of the amino-truncated NOV proteins.

### The expression of the amino-truncated form of NOV and the chemoresistance of bladder cancer

**Figure 5.**
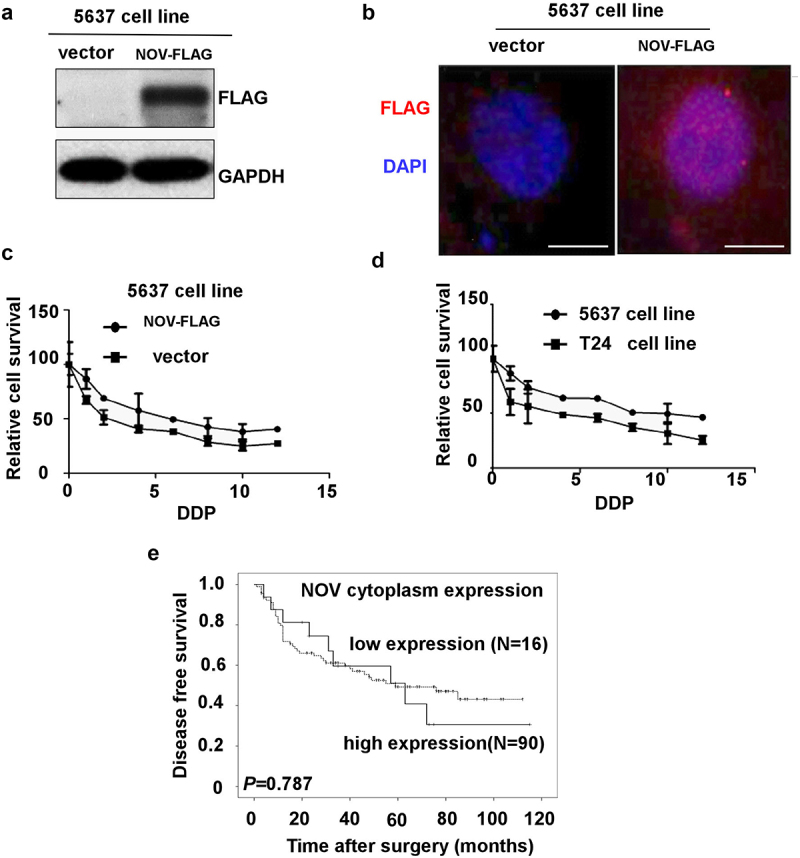
The over-expression of amino-truncated NOV enhances chemoresistance. (a–b): over-expression of amino-truncated NOV (NOV^188–356^, NOV-FLAG) in 5637 bladder cancer cell line. (c–d): chemoresistance between different bladder cancer cells. (e): disease-free survival analysis of 106 non-muscle invasive bladder cancer patients with different cytoplasm NOV protein expression. (Log-rank test: *p* = .787).

The amino-truncated variants of NOV are localized to the nucleus, and the carboxy-terminal (CT) module of NOV is responsible for that nuclear localization. The over-expression of truncated NOV in 5637 cells was verified using western blot analysis (Figure 5(a)). Immunofluorescent staining revealed that the truncated NOV was mainly expressed in the nuclei of 5637 cells, in line with the previous report (Figure 5(b)). We investigated the influence of the expression of truncated NOV on chemotherapeutic resistance. The 5637 bladder cancer cell line overexpressing the amino-truncated NOV and control cells was treated with different concentrations of cisplatin (DDP) (0, 1, 2, 4, 6, 8, 12 μmol/L) for 48 h. The 5637 cells overexpressing the truncated NOV protein had an enhanced response to treatment with DDP (Figure 5(c, d), and the IC50 values of the two groups were 6.068 μmol/L and 2.474 μmol/L, respectively. We also found the same results in T24 cells, which have a higher level of expression of the truncated form of NOV compared with the level in 5637 cells. Using immunochemical staining, we then examined the expression of the amino-truncated form of NOV in bladder cancer samples from patients who had received intravesical chemotherapy. In all, 106 patients with NMIBC underwent regular intravesical chemotherapy with mitomycin (MMC) or epirubicin (EPI) following TURBT. With a median follow-up time of 58 months, the 2-y DFS of groups with high and low expression levels of NOV^188-356aa^ were 55% and 72%, respectively (*p* < 0001). This result implies that NOV^188-356aa^ expression is associated with the chemoresistance of bladder cancer. While the DFS of groups with high and low expression levels of cytoplasm NOV (full-length NOV) were not significantly different (Figure 5(e)).

### Impact of the amino-truncated form of CCN3 expression on the proliferation, migration and invasion of bladder cancer cells

**Figure 6.**
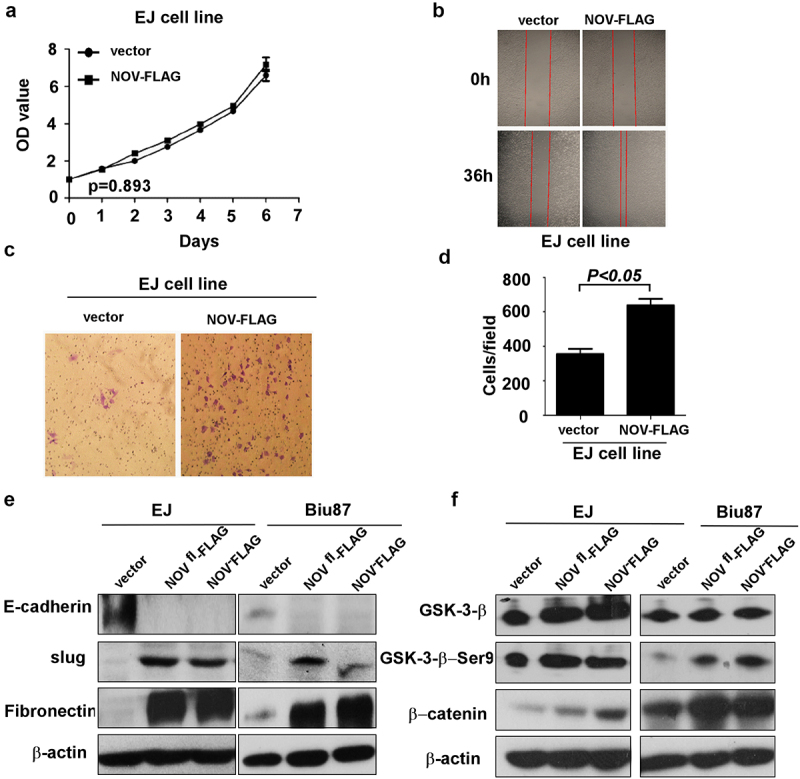
The over-expression of amino-truncated NOV (NOV^188-356^) enhances migration of bladder cancer cell. (a): the cell growth rates of different cells (b): the migration ability measured by wound-healing assay (c–d): the invasive ability measured by matrigel invasion chamber assay (e): over-expression of amino-truncated NOV (NOV^188-356^,NOV-FLAG) increases fibronectin and slug, inhibits E-cadherin. (f): over-expression of amino-truncated NOV (NOV^188-356^,NOV-FLAG) increases GSK-3β, GSK-3β-Ser9 and β-catenin.

To further investigate the impact of the truncated NOV protein on proliferation, invasion and metastasis in bladder cancer cells, the NOV^188-356aa^ expression plasmid was stably transfected into the bladder cancer cell line EJ. The cell viability assay (MTT assay) showed that the truncated NOV did not affect the proliferation of bladder cancer cells (Figure 6(a)). The impact of the truncated NOV on the migration of bladder cancer cells was examined by a wound-healing assay. The overexpression of truncated NOV enhanced the migration of bladder cancer cells compared with the migration of the controls (Figure 6(b)). A Matrigel invasion chamber assay was then performed to determine the effect of the truncated NOV on the invasion of bladder cancer cells. The results showed that the overexpression of truncated NOV enhanced bladder cancer cell invasion compared to invasion by the respective control cells (Figure 6(c, d)). We propose that the nuclear localization of the amino-truncated NOV proteins is correlated with the oncogenicity and chemoresistance of bladder cancer cells.

### EMT and the Wnt/β-catenin signaling pathway in amino-truncated nov-regulated cell migration, invasion and chemoresistance of bladder cancer cells

A key mechanism responsible for the invasiveness and metastasis of various cancers is EMT. The increase in expression of the amino-truncated form of the NOV protein enhanced the aggressive metastatic phenotype in bladder cancer, perhaps in part because of EMT. As shown in Figure 6(e), the overexpression of the amino-truncated form of the NOV protein in EJ and Biu87 cells decreased the expression of E-cadherin epithelial markers and concomitantly enhanced the expression of fibronectin mesenchymal markers. Furthermore, the protein level of the slug was enhanced after the increase in the level of the amino-truncated form of NOV (Figure 6(e)). To determine the possible signaling pathways involved in the effects of NOV^188-356aa^ in bladder cancer, western blot analysis was used to assess the effect of NOV on the serine phosphorylation of GSK-3β and β-catenin in EJ and Biu87 cells. The results indicated that the serine phosphorylation of GSK-3β and β-catenin was increased in EJ cells overexpressing the amino-truncated form of NOV compared with the level of phosphorylation in control cells (*p* < .01). Serine phosphorylation of GSK-3β and β-catenin was also enhanced in Biu87 cells overexpressing NOV^188-356aa^ compared to the level of phosphorylation in control cells (*p* < .001) (Figure 6(f)). These results suggest that the expression of the amino-truncated form of NOV is potentially responsible for enhancing the progression and chemoresistance of bladder cancer by inducing EMT and enhancing the serine phosphorylation of GSK-3β and β-catenin.

## Discussion

The function of NOV expression has been reported in several cancers, such as prostate cancer, CML, colorectal cancer, melanomas and bladder cancer.^10,11,13–15^ Chen reported that the NOV protein enhanced the migration of prostate cancer cells by increasing ICAM-1 expression. Reduced NOV expression was correlated with disease progression in colorectal cancer and was associated with the survival, invasion and chemoresistance of cancer cells.^14^ Overexpressed NOV matricellular protein regulated integrin expression, adhesion, and dissemination in melanoma cells.^11^ Chen et al. demonstrated that NOV might promote carcinogenesis in bladder cancer via promotion of EMT and association with increased mTOR activity.^10^ Most of these reports were concentrated on the function of the full-length NOV protein, while the presence and function of the nuclear truncated NOV in tumors were rarely reported. However, in most of the existing studies, the amino-truncated NOV protein was reported to be associated with tumorigenesis and the regulation of transcription.^22,27^ It was localized to the cell nucleus, and the CT module directed this process as a nuclear localization signal (NLS). As reported, this variant can inhibit transcription in mammalian cells, such as PAI-2/SerpinB2.^29^ PAI-2 acts as a tumor repressor gene in several cancers, such as hepatocellular carcinoma, pancreatic cancer and NSCLC.^30–32^ Although the amino-truncated NOV protein was detected years ago, its sequence was not defined until now, and the role of NOV^188-356aa^ in cancers was unknown.

In this study, the expression of the NOV protein was up-regulated in bladder cancer tissue compared to the levels in adjacent normal bladder tissue. High levels of nuclear NOV protein had a significant relationship with the histopathological classification of patients, the T stage, lymph node metastasis and recurrence. The up-regulated expression of the nuclear NOV protein predicted worse survival rates and poor prognoses. Furthermore, the multivariate Cox regression analysis showed that a high nuclear expression level of the NOV protein was an independent predictor for patient survival with bladder cancer. In non-muscle-invasive bladder cancer, there were significantly lower OS and DFS rates in the group with high nuclear expression levels than the rates in the group with low nuclear expression of the NOV protein. These results indicated that the nuclear expression of the NOV protein may be a predictive marker for disease progression and the risk of recurrence in bladder cancer, including non-muscle invasive bladder cancer, which was treated with TURBT and intravesical chemotherapy.

By applying matrix-assisted laser desorption ionization-time of flight MS to the purified 26 kDa NOV variant, we determined the exact sequence of this truncated protein and found that the truncated NOV/CCN3 protein is cut at the 187th amino acid of the full-length protein found in bladder cancer, causing it to lose its signal peptide, IGFBP and VWC domains. Furthermore, the enzymatic activity and the expression of MMP3 contributed to the formation of the amino-truncated NOV proteins.

To expand our understanding of the role played by the amino-truncation of NOV proteins in bladder cancer, we examined the influence of the amino-truncation of the NOV protein on the cellular functions of bladder cancer cell lines. The NOV protein inhibited the proliferation of cancer cells in malignant adrenocortical tumor cells and glioblastoma,^33,34^ while, NOV promoted proliferation and invasion of gastric cancer cells.^35^ In our study, the truncated NOV protein did not affect the proliferation of bladder cancer cells, but it enhanced the migration and invasion of bladder cancer cells. This indicated that the expression and function of NOV in malignancies may be organ- or tissue-specific.

In prior studies, fl-NOV was associated with tumor development, invasion and migration in some malignancies, while in other cancers, it acted as a tumor repressor gene.^9,11,15,17^ Amino-truncated NOV proteins were reported to be correlated with the progression of cancer.^27^ In this study, the truncated variant of the NOV protein lost its N-terminal peptides, including the signal peptide, IGFBP and VWC, and then NOV^188–356^ was localized in the nucleus, playing an important role in bladder cancer. The TSP1 and CT domains were conserved NOV.^188–356^ Compared to other members of the CCN family, the TSP1 and CT domains of NOV^188–356^ were similar to CCN4–6, which are involved in the Wnt pathway. Therefore, we examined whether the migration, invasion and chemoresistance of bladder cancer cells expressing NOV^188–356^ were also activated through the Wnt pathway. Interestingly, the expression of NOV^188–356^ was associated with EMT and Wnt pathway in vitro experiment.

Our present study suggests that the amino-truncated NOV protein plays a pro-tumorigenic role in bladder cancer, and it is up-regulated in bladder cancer. In contrast to prior studies, the truncated NOV protein (NOV^188–356^) was an independent predictor of OS and DFS rates, unlike the cytoplasmic NOV (fl-NOV) expression, which was investigated in prior studies.^10^ In NMIBC patients who were treated with intravesical chemotherapy following TURBT, higher expression levels of NOV^188–356^ were correlated with lower rates of disease-free survival. These clinical results implied that NOV188–356 might be involved in chemoresistance in bladder cancer. Consequently, in in-vitro experiments, overexpression of NOV^188–356^ was correlated with chemoresistance of cisplatin in bladder cancer cells. The mechanism underlying these processes might rely on the activation of the Wnt pathway and EMT in bladder cancer.

## Supplementary Material

supplementary clean.docx

## Data Availability

The authenticity of this article has been validated by uploading the key raw data onto the Research Data Deposit public platform (www.researchdata.org.cn), with the approval RDD number as RDDB2018000249.
