# Supplementary material for: Amino-truncated NOV expression and its correlation with clinicopathologic features, prognosis, metastasis, and chemoresistance in bladder cancer
Source: Cancer Biol Ther. 2024 Aug 3;25(1):2386753. doi: 10.1080/15384047.2024.2386753 (PMC11299625; doi:10.1080/15384047.2024.2386753)
Supplement: supplementary clean.docx [file KCBT_A_2386753_SM3972.docx]

***Immunohistochemistry analysis***

Immunohistochemistry (IHC) was performed according to instruction [1]. The 4-μm paraffin-embedded sections of bladder cancer tissues were prepared for immunohistochemistry. The rabbit polyclonal anti-NOV antibody (1:400, Abcam, Cambridge, UK) was used. Blinded to the clinical outcome, the results of the staining were scored by 2 pathologists independently. One score is given according to the intensity of staining as: negative (1 point); weak (2 point); moderate (3 point); strong (4 point). Another score is given according to the percentage of positive cells as: ≦25% of the cells (1 point); 26%-50% of cells (2 point); 51%-74% of cells (3 point); ≧75% of cells (4 point); A final score is calculated by multiple the 2 scores. If the final score is bigger than 4, the NOV expression is considered high expression; otherwise, it is considered low.

***Real-time PCR and Western blot analysis***

Total RNA was isolated from cell lines and tissues using Trizol reagent (Invitrogen, Grand Island, NY) and reversely transcribed using the reverse transcriptase cDNA synthesis kit (Fermentas, St Leon-Rot, Germany) according to the manufacturer's instructions. The cDNA was then used as a template in real-time PCR. The accession numbers for NOV and GAPDH gene sequences used for primer construction were NM_002514.4 and NM_001357943.2.NOV upstream primer: 5 '-GCATCTGCACGGCGGTAG-3', and downstream primer: 5 '-CACTGGAATTTGCAGCTTGG-3'. Upstream primer of GAPDH: 5 '-CTCCTCCTGTTCGACAGTCAGC-3', downstream primer: 5 '-CCCAATACGACCAAATCCGTT-3'. Quantitative PCR was performed by using SYBR Green I Kit (Takara, Dalian, China) in ABI PRISM 7500 Sequence Detection System (Applied Biosystems, Foster City, CA). Real-time PCR reaction procedure: 1) 50℃ for 2 min; 2) pre-denaturation at 95℃ for 10 min; 3) denaturation at 95℃ for 15 s, annealing and extension at 60℃ for 1 min, 40 cycles. The relative quantitative experiment adopted CT value comparison method and GAPDH as internal reference.

Western blot analysis was performed as usual and the Western blots were probed with rabbit polyclonal anti-NOV antibody (1:1000, Abcam,Cambridge,UK,ab137677), anti-GAPDH (Sigma,1:3000,Mouse, G8795), anti-β-actin (BD, 1:3000, Mouse 612657), anti-Flag (Sigma,1:1000,Mouse, F1804), anti-HIS (BD,1:1000,Mouse, 552565), anti-MMP3 (Abcam, 1:1000, Mouse, ab268084), anti-β-catenin (BD,1:1000,Mouse, 610153), anti-Vimentin (BD,1:1000,Mouse, 550513), anti-α-tubulin (Sigma, 1:3000, Mouse, T6199 ), anti-Fibronectin (BD, 1:1000, Mouse,610077), anti-E-Cadherin (BD, 1:1000, Mouse, 612131), anti-slug (BD,1:1000, mouse,564614 ), anti-Total GSK-3β (BD, 1:1000, mouse, 610201 ), anti-p GSK-3β-Ser9 ( Cell Signaling Technology, 1:1000, 9336S ).

***Immunofluorescence Analysis***

BIU87 Cell lines were plated on culture slides (Costar, Cambridge, MA) for 24h and then rinsed with phosphate-buffered saline (PBS) and fixed in paraformaldehyde for 15 min at room temperature. The cells were then blocked for 30 min in 10% BSA (Sigma-Aldrich St. Louis, MO) in PBS and then incubated with polyclonal anti-NOV antibody in BSA for 12h at 4℃. After three washes in PBS, the slides were incubated for 1h in the dark with FITC-conjugated goat anti-rabbit IgG (1:1000) (Jackson Laboratory). After three washes, the slides were stained with 0.5 μg/mL 4-,6-diamidino-2-phenylindole (1 mg/mL stock prepared in deionized water; DAPI; Sigma-Aldrich St. Louis, MO) for 5 min to visualize the nuclei, and examined using an Olympus confocal imaging system (Olympus FV100) (oil immersion). Images were then analyzed via the Olympus FV 1000 software (Olympus, Fluoview, version 1.7, Tokyo, Japan).

***N-Terminal Microsequencing***

Recombinant NOV188-356aa protein expressed by Hi5 baculovirus-infected cells was concentrated from cell culture supernatant with 10kd ultrafiltration tube. Then it was purified to homogeneity using Ni-NTA and flag-M2-beads. In Ni-NTA wash steps, buffers with an imidazole concentration of 50 mM were used. Absorpted proteins eluted with the wash buffer containing 50mM imidazole were collected. For flag-M2-beads, flag-peptide solution was used to collect proteins absorpted on it. Both 50mM imidazole and lag-peptide solution were concentrated by 10kd ultrafiltration tube again.

Then concentrated samples were subjected to 7.5% SDS/PAGE. Both protein bands were excised and subjected to MALDI-TOF-MS and N-terminal amino sequencing.

***Caesin zymography***

Samples were submitted to electrophoresis under nonreducing conditions on 7.5% SDS-polyacrylamide gels copolymerized with 0.1% w/v casein sodium salt. When the electrophoresis was finished, activation of MMP pro-forms was achieved with 2 mM p-amino phenylmercuric acetate (APMA) for 1 h at 37 °C. The gels were incubated at 37 °C for 24 h in zymography incubation buffer (CaCl_2_, 5 mM, NaCl, 150 mM and Tris-HCl, 50 mM, pH 8.0). Negative control zymograms were incubated in 5 mM ethylenediamine tetracetic acid (EDTA) and 2 mM 1, 10-phenanthroline to inhibit MMP-3 isoforms. Then the gels were stained in 0.2% Coomassie Brilliant Blue R-250 and destained.

**Reference**

1. Qin ZK, Yang JA, Ye YL, Zhang X, Xu LH, Zhou FJ, Han H, Liu ZW, Song LB, Zeng MS. Expression of Bmi-1 is a prognostic marker in bladder cancer. BMC Cancer 2009; 9: 61.
